# Editorial: Moving beyond tokenism: toward sustainable youth engagement to improve the implementation of public health programs and interventions

**DOI:** 10.3389/fpubh.2025.1576788

**Published:** 2025-03-06

**Authors:** Ucheoma Catherine Nwaozuru, Emily Ruth Haines, Thembekile Shato, Chisom Obiezu-Umeh, Juliet Iwelunmor

**Affiliations:** ^1^Department of Implementation Science, Wake Forest University School of Medicine, Winston-Salem, NC, United States; ^2^Department of Surgery, Division of Public Health Sciences, Washington University in Saint Louis School of Medicine, St. Louis, MO, United States; ^3^Department of Medical Social Sciences, Center for Dissemination and Implementation Science, Northwestern University Feinberg School of Medicine, Chicago, IL, United States; ^4^John T. Milliken Department of Medicine, Division of Infectious Diseases, Washington University in Saint Louis School of Medicine, St. Louis, MO, United States

**Keywords:** youth engagement, implementation science, programs, youth–young adults, participatory

## Introduction

Youth engagement involves the leadership of adolescents and young adults in developing, implementing, and evaluating programs and research that impact their health and well-being (1). Youth engagement in its true form puts youth at the center of research by shifting the power dynamic between researchers and youth participants. A growing body of evidence demonstrates the benefits of youth engagement in bridging the research-to-practice gap for youth-based interventions (2–4). Thus, it is increasingly considered essential to involve individuals with lived experience in all stages of research projects and to examine issues relevant to them to improve the quality and relevance of research.

This Research Topic, “*Toward Sustainable Youth Engagement to Improve the Implementation of Public Health Programs and Interventions*,” was developed to document collective efforts to promote youth engagement in implementing public health programs and interventions. It is a compilation of articles that share insights, strategies, and lessons learned from various research projects and initiatives that have successfully engaged youth in public health. While not exhaustive of the work in this area, these articles reflect the growing importance of youth engagement in research, some approaches to youth engagement, and lessons learned. We summarize the articles in this Research Topic under two main and overlapping themes:

## Youth engagement to identify salient needs and solutions for health issues impacting youth

Ratliff et al. led this Research Topic by documenting lessons learned from their work focused on using *Youth Participatory Action Research (YPAR*) to engage young people who have experienced homelessness in Boston in research that impacts their lives. YPAR attributes youth disenfranchisement in part to adults' dismissal of their agency and challenges this by positioning youth as researchers who can shape study design and methods. Through YPAR, youth in this project generated research questions and were fully incorporated into the research team. They were trained to actively participate in research activities and were compensated for their time. Ratliff et al. commented on the challenges faced in engaging a uniquely vulnerable population of young people in YPAR and offered practical solutions for fostering inclusive, trauma-responsive, and flexible environments to mitigate these challenges. Similarly, Chow et al. highlight *youth community advisory boards (CABs)* as sustainable mechanisms for youth engagement in developing policies and advancing the implementation of interventions for young people. In the manuscript, the authors described a network of youth CABs comprising young people between the ages of 13–26 years living with or affected by HIV operating in four regions of Tanzania. The CABs were engaged to understand the priority health needs of Tanzanian youth and to further generate youth-driven solutions to address these needs. Chow et al. highlighted the importance of youth engagement in setting policy agendas to ensure that the needs of youth are met. The study underscores the relevance and importance of actively engaging youth in addressing issues that affect them. Overall, the two studies emphasize the need for youth engagement not only to identify the problems that affect youth and their proposed solutions but also to offer strategies for engaging them in subsequent phases of research design and implementation.

## Co-creation strategies to foster youth engagement in research

Bennin et al. leveraged a youth research group (YRG) model to guide, co-create, monitor, and evaluate the implementation of PrEP delivery to young people (15–29 years old) in Cape Town, South Africa. The YRG was a youth group convened to guide the intervention development, implementation, and evaluation process. The YRG comprised 80 young people aged 16–29, purposively selected to represent the population of interest. An evaluation of the model garnered positive feedback from the YRG members, which included feelings of empowerment, a perception of genuine youth engagement, and consultation that was not tokenistic. Nwaozuru et al. used an innovation bootcamp model to engage youth in developing and implementing HIV self-testing delivery strategies for youth in Nigeria. The innovation bootcamp, informed by human-centered design principles, is an accelerated training program to provide research implementation, entrepreneurial skills, and project management skills among participants. The manuscript describes the innovation bootcamp process and the five youth-driven HIV service delivery strategies that emerged. The study highlights the feasibility of the innovation bootcamp model in building the ability of youth to lead solutions and the potential to increase the sustainability of community interventions. In addition, Coakley et al. described the use of Youth Participatory Action Research (YPAR) to engage adolescents and young women in the design and delivery of a sports-based, after-school sexual and reproductive health program in South Africa. This methodology involved multiple steps, such as youth investigator recruitment, youth investigator training, co-design of YPAR methods, youth investigator-led data collection, collaborative analysis and interpretation, and dissemination. The evaluation of the process highlighted high program acceptability, in part due to the inclusion of end users- adolescent girls and young women- in the design, delivery, and evaluation of this intervention. Collectively, these studies provide descriptions of innovative models for including youth in the design, delivery, and evaluation of interventions. In addition, the studies also highlight the need to further explore sustainable and creative strategies for continued youth engagement beyond the duration of the intervention implementation and evaluation process.

## Conclusion

In conclusion, this Research Topic is an attempt to collect lessons and recommended practices from researchers on how to foster meaningful youth engagement and avoid tokenism. The articles included in this Research Topic highlight the benefits and utility of youth engagement and some strategies for youth engagement. They also showcase different levels of involvement, with some studies integrating youth in multiple phases of the research process, such as research question generation, study design, data collection, analysis, implementation, evaluation, and dissemination. This underscores both the feasibility and the value of youth participation in all aspects of research. While the level of engagement may vary based on feasibility, the studies in Research Topic —primarily conducted in African countries—offer valuable insights that contribute to the broader discourse on youth involvement. While the applicability of these findings may have regional limitations, they provide important perspectives on how to sustain meaningful youth engagement. We hope that this Research Topic will spark further discussion and actions to ensure that youth voices are front and center in issues that affect them.
